# Missed opportunities in mixed methods EdTech research? Visual joint display development as an analytical strategy for achieving integration in mixed methods studies

**DOI:** 10.1007/s11423-023-10234-z

**Published:** 2023-05-11

**Authors:** Mitchell Peters, Sergi Fàbregues

**Affiliations:** 1 Office of Research & Innovation, Universitat Oberta de Catalunya, Barcelona, Spain; 2 Faculty of Psychology and Educational Sciences, Universitat Oberta de Catalunya, Barcelona, Spain

**Keywords:** Mixed methods research, Mixed analysis, Visual joint displays, Educational technology, Meta-joint display

## Abstract

Mixed methods research is becoming more prevalent in educational technology due to its potential for addressing complex educational problems by integrating qualitative and quantitative data and findings. At the same time, a growing chorus of researchers laments the quality and rigor of research in this field. Mixed methods studies which demonstrate explicit integration in educational technology research are scarce, and even fewer apply integration strategies recommended in the literature, such as visual joint displays. Failure to address the challenge of comprehensive integration may result in missed opportunities for deeper insights. To address this methodological problem, the purpose of this paper is to shed light on the procedures, opportunities, and practical challenges associated with mixed methods integration through the use of visual joint displays as an analytical tool for data interpretation and reporting in these types of designs. Using an exploratory sequential mixed methods multiple case study design as an illustrative example, we will (1) provide step-by-step guidance on how to develop a visual joint display to conduct an integrated analysis in a complex mixed methods design; (2) demonstrate how to use a display of this type to integrate meta-inferences previously generated through a series of interconnected joint displays; and (3) illustrate the benefits of integrating at the literature review, theoretical, analysis, interpretation, and reporting levels in mixed methods studies. This methodological article aims to advance knowledge in educational technology research by addressing the integration challenge in mixed methods studies and assisting researchers in this field in achieving comprehensive integration at multiple levels.

## Introduction

Educational Technology (EdTech) is an expansive and dynamic research field with a global scope. The COVID-19 pandemic has brought greater public attention and academic scrutiny to the influence of EdTech at all stages of formal education and lifelong learning, including criticism of the quality and rigor of EdTech research. Critical commentary on the methods used to solve complex educational problems remains an important and consistent debate. Systematic mapping of research patterns has become a lively and well-established research area (Bozkurt, 2020; Reeves & Lin, 2020; Reeves & Oh, 2017) and common concerns in the literature include ambiguity and confusion toward how best to approach methodological design (Peters et al., 2022), a lack of theoretical development (Hew et al., 2019), and a tendency for basic forms of descriptive research (Bulfin et al., 2014) where researchers seek out evidence of ‘what works’ when technology is applied in the classroom (Castañeda & Williamson, 2021). Motivated by the added value gained by integrating qualitative and quantitative methods or approaches (Bazeley, 2018; Creamer, 2018; Creswell & Plano Clark, 2018; Fetters & Molina-Azorin, 2017), educational researchers are increasingly choosing mixed methods research (MMR) designs and procedures, yet transparent examples or clear guidelines to support integration in these types of designs are scarce, particularly in the EdTech field (Bustamante, 2019). In this context, joint displays can provide an enormous opportunity for enhancing integration by facilitating the systematic synthesis of quantitative and qualitative data and the production of integrated interpretations of MMR findings (Bustamante, 2019; Fetters & Guetterman, 2021; Peters et al., 2021). Despite a growing literature on emerging techniques for joint display development (Fetters & Guetterman, 2021; Guetterman et al., 2015; Haynes-Brown & Fetters, 2021; Younas & Durante, 2022), there is a scarcity of EdTech research which illustrates the iterative process of constructing and employing displays of this type as analytical tools.

The objective of the current paper is to describe in detail the procedures, opportunities, and practical complexities associated with integrating quantitative and qualitative data within MMR designs using visual joint displays as tools for data analysis, interpretation, and reporting. We will illustrate the process using a published example from the EdTech field linked to guidelines in the MMR literature. Despite their growing relevance, most MMR integration procedures in EdTech research lack sophistication, are often underdeveloped, and therefore represent missed opportunities for gaining new understandings of complex phenomena. Considering this problem, we aim to shed light on the integration process and bring transparency to this much discussed methodological challenge in the MMR field (Fetters & Molina-Azorin, 2017; Plano Clark, 2019).

We intend to contribute to the existing literature on joint displays in MMR and extend what is already known about this methodological approach in the EdTech field in three ways: (1) by offering step-by-step guidance on the process of visual joint display construction as a method to conduct an integrated analysis in a complex MMR design, as opposed to an approach merely for reporting results; (2) by illustrating how a visual joint display was used to integrate meta-inferences previously generated from a series of interconnected joint displays; and (3) by demonstrating the expansive range of purposes served by visual joint display construction to integrate at the literature review, theoretical, analysis, interpretation, and reporting levels. Additionally, we intend to discuss the analytical potential of joint displays which add value by incorporating robust theoretical frameworks into the research design, integration strategies and procedures for mixed analysis and reporting. This is particularly salient in EdTech research as it has long been characterized as an under-theorized field (Hew et al., 2019).

## Literature review

### MMR in EdTech research

MMR has emerged in the social and educational sciences as a particularly useful way of investigating complex phenomena that cannot be ideally investigated using a single method. MMR involves a number of study designs based on the integration of data collection, analysis, and inference techniques associated with quantitative and qualitative research approaches to generate a nuanced and comprehensive understanding (Creswell & Plano Clark, 2018; Johnson & Onwuegbuzie, 2007). Several methodological reviews in different disciplines reveal that MMR approaches can afford many potential advantages for answering complex questions (Howell Smith & Shanahan Bazis, 2021). These include using the strengths of qualitative and quantitative research to offset their respective limitations; increasing the breadth and depth of the study; and evaluating distinct, similar, or seemingly contradictory findings about a phenomenon (Fetters, 2020).

In the EdTech field, Poth (2018) and Ngulube and Ukwoma (2022) have argued that MMR is particularly useful for addressing the multidimensional nature of the types of complex problems that arise in teaching and learning environments in higher education. According to Poth (2018), gaining access to multiple perspectives on the phenomenon under investigation, improving the validity of evidence, and generating a number of mixed insights that would have been unattainable using a single method highlight “the contribution that mixed methods approaches can have in advancing educational technology in higher education” (p. 15). Similar observations about the capacity of MMR to address multifaceted problems and strengthen the state of knowledge of EdTech research have also been made by Peters et al. (2022) and Krumsvik (2020).

### Integration as a core component of MMR

Integration, a defining element of MMR studies and what distinguishes this type of research from monomethod and multimethod research (Åkerblad et al., 2021; Bazeley, 2018; Fàbregues & Molina-Azorin, 2017), is defined in the literature as the deliberate mixing of the quantitative and qualitative components throughout the research process with the aim of generating “a new whole or a more holistic understanding than achieved by either alone” (Fetters & Molina-Azorin, 2017, p. 293). To achieve this aim, researchers combine distinct quantitative and qualitative research elements in a study so that they become interdependent in order to achieve a common research purpose (Bazeley, 2018; Vogl, 2019). As a result of the synergy created during the integration process, researchers can improve their descriptions and interpretations of investigated phenomena, add more depth and texture to their answers to study research questions, and gain deeper insights that would not have been revealed otherwise (Bustamante, 2019; Fetters & Freshwater, 2015).

A consensus exists in the literature that integration is difficult due to the distinct nature of quantitative and qualitative approaches and the practical difficulties associated with “tying the two (approaches) together” (Bryman, 2007, p. 16). In response to these challenges, several authors (Bazeley, 2018; Bazeley & Kemp, 2012; Creamer, 2018; Fetters, 2020; Fetters et al., 2013; Pluye et al., 2018) have developed practical strategies to help researchers determine how and why to integrate, including defining the purpose of integration and guiding the practical aspects of the integration process. These strategies include, among others, building, connecting, exploring, comparing, and expanding. Building occurs when data from one component are used to inform the data collection and analysis of the following component. Connecting is similar to building, with the difference that the data from one component are used to determine the sampling strategy for the other. Exploring involves collecting and analyzing qualitative data to examine a concept prior to a follow-up quantitative study. Comparing entails examining the relationship between quantitative and qualitative data on a phenomenon of interest. Expanding implies using qualitative and quantitative data collection methods to develop an expanded but overlapping perspective on a phenomenon. In order to take advantage of the potential added value of MMR, researchers must be aware of these strategies (Fetters & Molina-Azorin, 2017; Younas & Durante, 2022). Indeed, one effective way of achieving this potential value is to use a joint display tool, that can take the form of a table, figure, or graph to facilitate the operationalization of the previously described integration strategies and, when appropriate, to articulate the meta-inferences that have resulted from this integration (Guetterman et al., 2015; McCrudden et al., 2021).

Despite the popularity of integration, MMR studies explicitly integrating quantitative and qualitative components are infrequent within the EdTech field, and even fewer employ the integration strategies and joint displays recommended in the MMR literature. As argued by Bazeley (2018), the failure to integrate is problematic because opportunities for a better understanding of the phenomena being studied will be missed, the potential for gaining depth and texture will be lost, and, in some cases, erroneous conclusions will be generated. Because inadequate MMR practices in EdTech can be a result of a lack of methodological training and guidelines, practical examples of integration can aid in the design and implementation of robust MMR investigations.

### Joint display analysis

Joint displays for integrating quantitative and qualitative data in MMR studies have gained increased attention in methodological publications and content-related empirical articles due to their transparency and structured approach to integrative thinking (Creamer, 2022; Guetterman et al., 2021; Plano Clark, 2019; Younas & Durante, 2022). These displays were not discussed in the MMR literature until the early 2010s, despite their value in facilitating the implementation of complex integrative analytic procedures and reporting the resulting outputs (Guetterman et al., 2015, 2021). Initial references to joint displays highlighted their usefulness as a reporting tool, but in recent years, MMR methodologists have begun to recognize that MMR joint displays can be used for more than simply conveying findings to readers.

Fetters and Tajima (2022) and McCrudden et al. (2021) describe three uses for joint displays: joint displays for representing the MMR findings in publications, joint displays for organizing MMR data collection and joint displays for facilitating MMR data analysis. The second of these variants, joint display analysis, is defined by Fetters and Guetterman (2021) as an iterative process in which researchers use the joint display structure to identify connections between qualitative and quantitative constructs and generate meta-inferences (i.e., integrated interpretations or conclusions derived from both the qualitative and the quantitative data) based on the ‘fit’ of the two types of data. Joint display analysis allows researchers to acquire new insights from those provided by the quantitative and qualitative components individually. It can thus be helpful during the execution of a study to facilitate the “cognitive process of merging, comparing, relating, and linking qualitative and quantitative data or results” (Guetterman et al., 2021, p. 1), allowing the researcher to achieve “integration and inferential transparency” (McCrudden et al., 2021, p. 1).

McCrudden et al. (2021) identified three main types of joint displays which can be applied to a range of research use cases in MMR studies: quantitative results matrix, integrated results matrix, and integrated visual display. Different joint displays can be applied and extended to different use cases in the expansive and varied field of EdTech research. For instance, quantitative results matrices allow researchers to identify individuals who vary from most of the sample in a systematic manner by juxtaposing quantitative findings. Integrated results matrices merge quantitative and qualitative findings to enable their comparison and the generation of meta-inference displays. Integrated visual displays represent the integration of quantitative and qualitative findings through visuals, such as graphs, images, or figures. While most early examples of joint displays included integrated results matrices of quantitative and qualitative findings, MMR researchers have innovated in recent years by developing integrated visual displays with various types of visuals, such as diagrams, figures, maps, and illustrations. According to Guetterman et al. (2021), visual joint displays can reduce the “cognitive burden” (p. 7) of the integration analysis and reporting processes compared to tabular joint displays. At the reporting level, they can make integration more explicit and facilitate the interpretation of complex integration findings. At the level of analysis, they can assist researchers in generating meta-inferences or making them more aware of the iterative development of the theoretical model utilized.

**Fig. 1:**
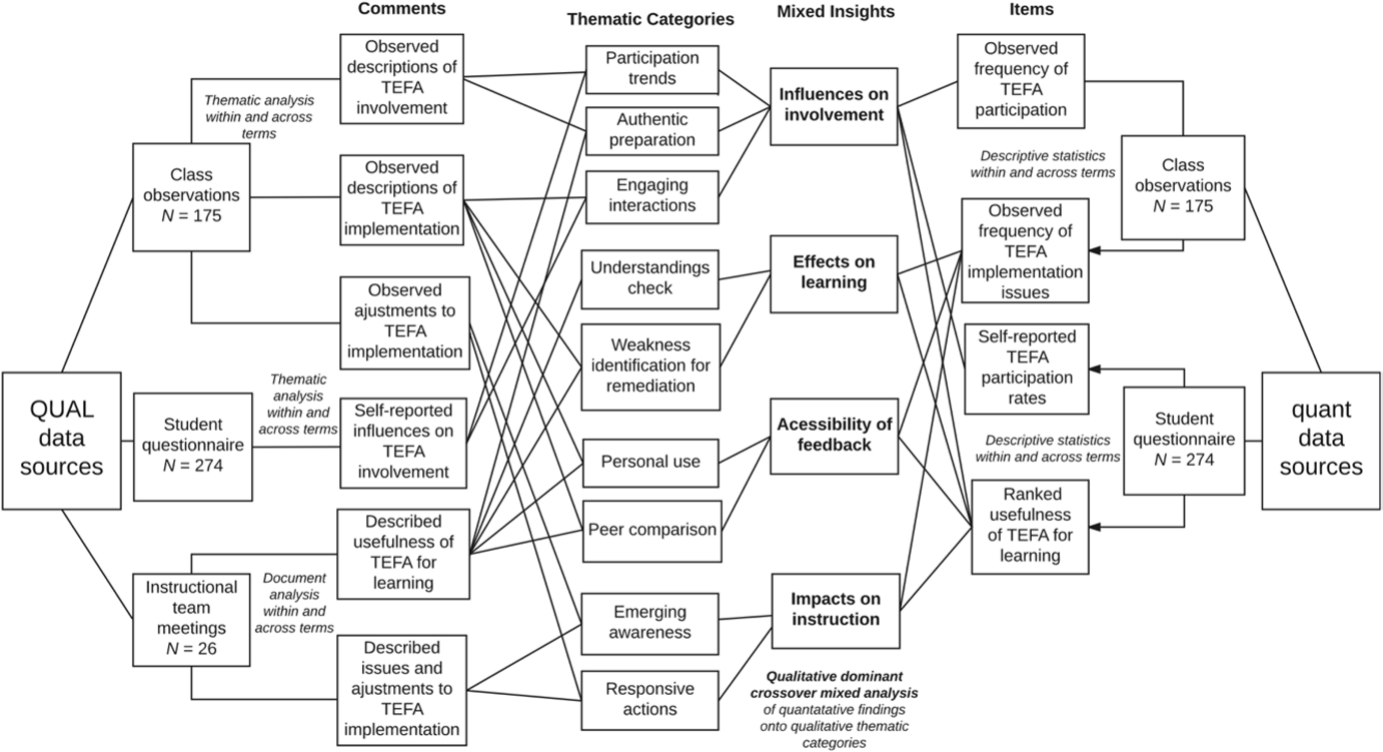
Visual joint display exemplar from Poth (2018)

An outstanding example of a visual joint display in the field of educational technology was presented by Bustamante (2019), who used a convergent design to evaluate a professional development program on Web 2.0 technologies for teachers of Spanish. Based on the Technological Pedagogical Content Knowledge (TPACK) theoretical model, the author developed a circular joint display made of several concentric rings representing, for each dimension of the TPACK model, the quantitative findings in the inner circle, the qualitative findings in the intermediate circle, and the integrated findings in the outer circle (i.e., an indication on whether the findings converge, diverge, or expand each other). Through this model, Bustamante (2019) was able to comprehensively integrate theory across all the study components. Another example in this field is Poth (2018), who carried out an embedded MMR case study design of two technology-enhanced formative assessment classroom strategies. Figure 1 presents the visual joint display developed by Poth (2018) with the qualitative data sources and thematic categories on the left, the quantitative data sources and items on the right, and the mixed insights emerging from integrating the quantitative findings with the qualitative thematic categories in the center. By triangulating qualitative and quantitative information regarding related components, Poth (2018) was able to gain sophisticated analytic insights and generate new synergistic understanding for each of the examined constructs.

Aside from Bustamante (2019) and Poth (2018), there are few examples demonstrating the use of visual joint display analysis in educational technology and other fields. However, recently, several publications have outlined the steps involved in this analytical MMR strategy. Haynes-Brown and Fetters (2021) described the iterations involved in creating a joint display with histograms in an explanatory sequential MMR study examining classroom teachers' use of technology. These authors described how additional rounds of analysis enabled them to reveal insights into the integrated findings by evaluating the *organizational intent*, *analytical intent*, and *effectiveness of the visuals* created in each iteration. Fetters and Guetterman (2021) and Guetterman and Fetters (2022) have published similar examples in the health sciences field. In this article, we expand on these previous examples by describing the process of creating a meta-joint display generated in a study examining lifelong learning in online higher education (Peters et al., 2021). Our example differs from previous examples in that it (1) combines two types of joint displays, namely, joint displays in the form of tables for analysis and joint displays in the form of visuals for reporting; (2) summarizes the themes identified in multiple joint displays tables used for mixed analysis into a final integrated meta-joint display; and (3) uses the radial display to link the findings with a theoretical model from the literature.

## Overview of illustrative research design

The empirical demonstration of mixed analysis and joint display construction derives from a three-year study on student experiences of lifelong learning in online postgraduate education, funded by the Universitat Oberta de Catalunya (UOC) (Peters & Romero, 2019; Peters et al., 2021). With the aim of increasing the breadth and depth of understanding of student experiences, we implemented an exploratory sequential MMR design, using a multiple-case study approach (Creswell & Plano-Clark, 2018). In these types of designs, the purpose of development is achieved by using the qualitative findings of the first component to inform the quantitative data collection and/or sampling strategies of the second component, and the purpose of complementarity is accomplished by integrating the findings of both components during the interpretation phase. The case studies included three postgraduate EdTech program populations at the UOC, the University of Illinois Urbana-Champaign, and the University of Edinburgh. At each site, four students were selected (12 in total) through purposive, criteria, and convenience sampling. An MMR design motivated by a complementarity purpose allowed the research team to examine different facets of the same phenomenon through different methods. These design decisions were considered a meaningful and in-depth way to advance theory related to emerging and networked forms of digital learning as well as enhance the usefulness of research findings.

The first qualitative component was used to explore the phenomenon from the participants’ perspective and to develop a quantitative survey instrument. Because no instruments existed to examine learning across formal and informal contexts, the qualitative findings informed questionnaire development using an MMR integration strategy at the research design level known as *building* (Fetters et al., 2013). Prior to data collection, an LE sensitizing model was built, following a sensitizing model technique (Van Den Hoonaard, 2008) to prepare potential lines of inquiry and developmentally shape the qualitative research instruments, including interview protocols, observation, and program documentation. The second quantitative component was a necessary design feature in order to complement the qualitative strand by collecting survey data from the broader population of students at each site through an online questionnaire (n = 178). A particular challenge in this design was building a quantitative survey as no previous instrument existed. The objective of the survey was to capture student attitudes and behavior of online learning across contexts—from formal to informal. The survey was built from the initial results of the thematic analysis and informed from the literature, informed by the three core components of the developed LE sensitizing model, and validated through using both content and face validity (Peters, 2019).

MMR integration procedures occurred at different phases of the study, including at the design level, and through mixed analysis at the interpretation and reporting levels. Mixed analysis was used to construct a meta-joint display, defined as the linking of two or more joint displays into a single figure (Fetters & Guetterman, 2021). Because of their complexity, meta-joint displays often require uncertain design decisions and thus reliance on established methodological guidelines. We relied on a data integration procedure known as a pillar integration process (PIP) to bring together data and draw insights from the qualitative and quantitative components (Johnson et al., 2019). The most striking challenge for the research team when conducting rigorous integration procedures was a lack of experience and training as well as access to clear guidelines for joint visual display development suitable for the field of EdTech. Uncertainty and doubt of whether the research process and associated results will be accepted by the research community (supervisors, peers, journal editors etc.) is a further challenge. The added value of using an MMR design was the development of a theory-driven and theory-advancing meta-joint display capable of revealing meta-inferences and new insight, interpretation and theory construction to the phenomenon under study, a technique seldom seen in the EdTech literature. The following section will describe in detail the iterative procedures involved in mixed analysis and meta-joint display construction.

## Overview of mixed analysis and iterative meta-joint display development

**Fig. 2:**
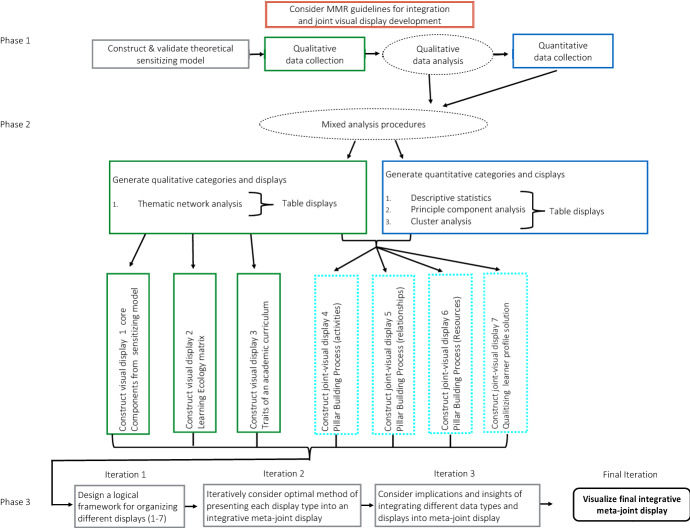
Procedural diagram for mixed analysis and visual joint display development. *Source* Author

Constructing novel meta-joint displays is a complex and iterative process, as outlined by Fetters and Guetterman (2021) and Haynes-Brown and Fetters (2021). Figure 2 outlines the identified phases of the illustrative case study. The current section will provide a step-by-step description of how a final meta-joint display was constructed by combining several interlinking joint displays through various integration strategies, namely, mixed analysis and reporting. Figure 2 highlights three distinct phases: (1) MMR guidelines and research design; (2) mixed analysis procedures; and (3) visual joint display iterations, and final integrated meta-joint display model. Phase 1 depicts the process of designing an MMR study, which implies the interconnected relationship between research design, integration strategies, and the type of joint display to be developed while carefully considering MMR guidelines. Phase 2 depicts the process of constructing and using visual joint displays to facilitate mixed analysis, while Phase 3 illustrates the iterative process of building a meta-joint display for representing MMR findings.

### Phase 1: MMR guidelines and research design

#### MMR guidelines and considerations

There has been little discussion in the EdTech methodological literature about integration procedures for mixed analysis and visual joint display construction. Therefore, reflexive, and critical engagement with the empirical EdTech and MMR literature is recommended in order to discover novel approaches for integrating qualitative and quantitative findings during analysis, such as Poth (2018), Bustamante (2019), Peters et al. (2021), and Haynes-Brown and Fetters (2021). Integration procedures at various levels in the current study were guided by Fetters et al. (2013), including at (a) the research design level; (b) the interpretation level through mixed analysis, and (c) the reporting level. Additionally, integration occurred at the theoretical and literature review level, as evidenced by the use of a theoretical sensitizing model. When we began our MMR study, we critically considered the interlinking relationship between research design, integration strategies at various levels, and the possible joint displays that can be constructed in response to mixed methods research questions.

Although MMR designs are increasingly used in EdTech research, there is some consensus that “meaningful integration of qualitative and quantitative data remains elusive and needs further development” (Guetterman et al., 2015, p. 554). Therefore, we considered established guidelines throughout all phases of the research process as recently outlined by Creamer and Edwards (2022) who distinguished guidelines for joint display development for analysis (*process*) and reporting (*product*) purposes by synthesizing recommendations from Guetterman et al. (2021) and McCrudden et al. (2021) available in Table 1. For example, we used a comprehensive theoretical framework to guide the research at all phases, including mixed analysis and meta-joint display construction.

**Table 1:** Guidelines for constructing visual joint displays for mixed analysis and reporting

Guidelines for mixed analysis	Guidelines for reporting
1. A robust theoretical or methodological framework	1. Self-explanatory
2. Creating a database, PowerPoint or spreadsheet that incorporates both qualitative and quantitative data	2. Avoids unnecessary complexity
3. Conducting rigorous intra-method analysis as a first step	3. Balances detail and synthesis
4. Leveraging visualization options available in qualitative software	4. Explained in the text
	5. Clarifies when and how integration occurred
	6. Recognizes inconsistencies in findings
	7. *Joint display* appears in the title of the display

*Source* Guetterman et al. (2021) and McCrudden et al. (2021)

Our mixed analysis phase was informed by the illustrative example of Bustamante (2019), who used the TPACK model. Although the current study used a less known theoretical framework, the EdTech field has a range of robust theoretical and conceptual frameworks to work from, including self-regulated learning, TPACK, technology acceptance model, and self-determination theory, among many others (Hew et al., 2019).

#### Sensitizing model development and exploratory sequential design

**Fig. 3:**
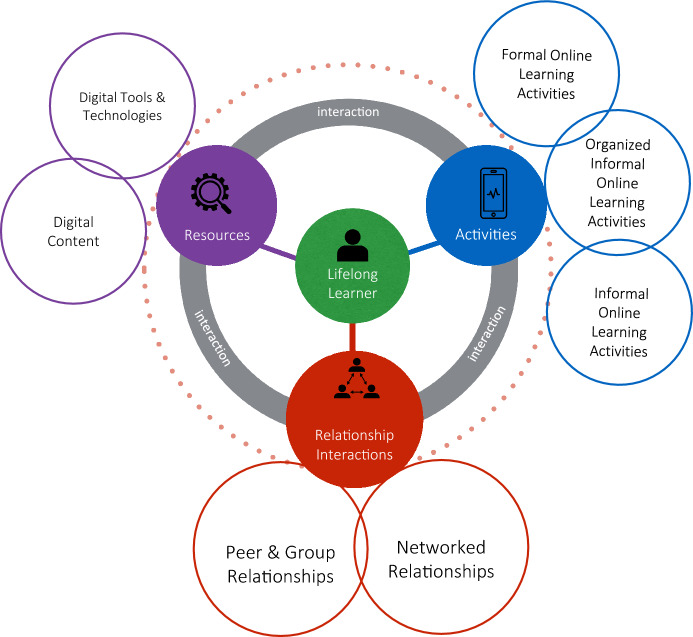
Learning ecology sensitizing model used for preparing potential lines of inquiry. *Source* Peters (2019)

Following an MMR exploratory sequential design, we constructed an initial Learning Ecology (LE) model following a sensitizing model technique (Van Den Hoonaard, 2008), seen below in Fig. 3. Sensitizing models are “derived from the research participants’ perspective, using their language or expressions, and that sensitize the researcher to possible lines of inquiry” (p. 1). The LE sensitizing model was designed prior to data collection as a guiding heuristic and organizing scheme, including preparing potential lines of inquiry such as being used to develop mixed methods research questions as well as initial interview and observation protocols. Further, it was used to guide thematic analysis, following a hybrid approach where theory-driven coding was generated directly from the core components and sub-components of the sensitizing model.

The LE sensitizing model was derived from the literature on lifelong learning, online higher education, and human development. The model was informed by Barron’s (2006) ontological definition, which identified learner activities, material resources, and social relationships as the core components of an individual’s learning ecology. The sensitizing model enabled researchers to define the qualitative and associated quantitative units of analysis, including (a) learner activities; (b) digital resources used to carry out core learner activities; and (c) relationship interactions and peer support. These units of analysis (known as core components of an individual’s LE) served to develop the qualitative data collection instruments, which included program documentation, in-depth interviews, and observation protocols. In-depth interviews allowed students to discuss experiences of postgraduate learning across contexts with an emphasis on the above-mentioned LE components.

Initial thematic network analysis of the qualitative data was informed by the LE sensitizing model following a hybrid coding approach and was used to develop the survey instrument from the quantitative component through *building* at the design level (Fetters et al., 2013). For example, the survey section related to formal and informal online activities was built from thematic categories generated in qualitative analysis of interviews, online observations, and program documentation, as well as from the digital competence framework for citizens (Vuorikari et al., 2016), which highlight the set of formal skills and knowledge required by individuals to productively learn, navigate, and participate in society. Respondents selected from a range of categorical items across three core sections: (1) learner activities; (2) digital resources used; and (3) relationship interactions to support academic learning. Using both descriptive and multivariate statistics, a principal components analysis (PCA) was used for data categorization and as a technique to reduce data complexity, draw inferences and yield conclusions from the survey data. Once rigorous intra-method analysis was completed, mixed analysis procedures could begin, detailed in phase 2.

### Phase 2: Mixed analysis procedures

When beginning the mixed analysis phase, integration of quantitative and qualitative findings was organized through tables, figures, and matrices. Logically, this implied in-depth familiarization with both types of data, allowing for the articulation and operationalization of the data so that the findings relative to equivalent quantitative and qualitative *constructs* could be compared (i.e., linkage) and, as a result, meta-inferences could be drawn (Fetters & Guetterman, 2021). Prior to mixed analysis, rigorous intra-method analysis was conducted to in order to operationalize both strands of results by generating categories and organizing displays which can link and compare equivalent qualitative and quantitative findings. For qualitative displays, we used thematic network tables to generate categories from the data, some of which were turned into matrix displays (see Fig. 3). The quantitative component used multiple tables to organize descriptive statistics, as well as the outputs from the PCA and cluster analysis solutions.

Visual display development required the use of software capable of visual design, such as Microsoft Power Point which was used as a common and accessible tool capable of collaborative review and feedback across a research team. Beginning with the LE sensitizing model, we built initial visual displays early in the research process in a slide deck, which also served as a visual research memo technique. A procedural diagram (see Peters et al., 2021), was also developed as a way to visualize integration at many levels, such as those at the design, procedures, interpretation and reporting levels. Visual displays were designed to evolve iteratively as the research unfolded, and as new insights and understanding were generated. The phase of mixed analysis was most concerned with the intention to better understand how the data were related to each other while looking for connections, including issues of commonality or discord.

**Fig. 4:**
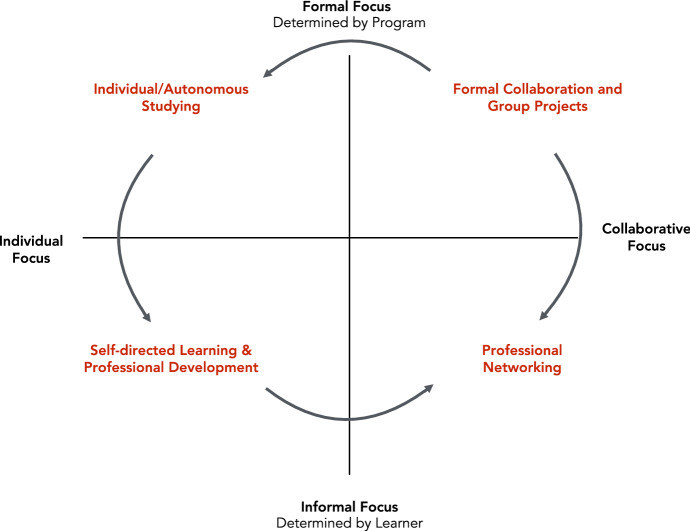
Learning ecology matrix in the context of online higher education. *Source* Peters, 2019

As a further step, we considered how best to visualize categories and displays through organizational intent. In the current study, seven displays were integrated to iteratively build a final meta-joint display model at different levels (Fig. 6), as demonstrated in the procedural diagram for mixed analysis in Fig. 1. Three visual displays derive from qualitative data sources, while four joint displays followed mixed analysis procedures. An example of a qualitative display (in contrast to a joint display) is shown in Fig. 4, where the organizing categories of a Learning Ecology matrix are presented, yielded through thematic analysis of the interview data characterizing student learning across a continuum of practices and contexts from formal and informal to collaborative and individual.

A pillar integration process, presented in Table 2, was integrated into the final meta-joint display. This process was selected among relatively few well-articulated integration techniques for MMR researchers where equivalent qualitative and quantitative constructs can be merged or interrelated in order to draw out meta-inferences (Johnson et al., 2019). On a pragmatic level, we followed Johnson et al’s. (2019) four stage outline for a PIP, completed sequentially after initial quantitative and qualitative analyses were concluded. Stage one included *listing* the raw data and associated categories for one strand, in our case quantitative, into columns in a table display. Stage two involved a *matching* process where qualitative strands were matched on the opposite side of the display and “﻿organized and compared across rows of the joint display so that the qualitative items reflect patterns, parallels, similarities, or any other relational quality with the quantitative items”(Johnson et al., 2019 p. 305). Stage three involved *checking* for the accuracy, completeness and quality of the match. Stage four involved *pillar building* in the final central column, comparing and contrasting the findings from the three previous stages in order to coneptualize new understandings indentified from merging and integrating the qualitative and quantitative columns. For example, the first pillar building theme in Table 2, *Core Information and Data Literacy Building Activities* was generated by going back and forth between the two types of data, allowing us to find commonality of constructs and themes congruent across the two interlinking data sources.

**Table 2:**
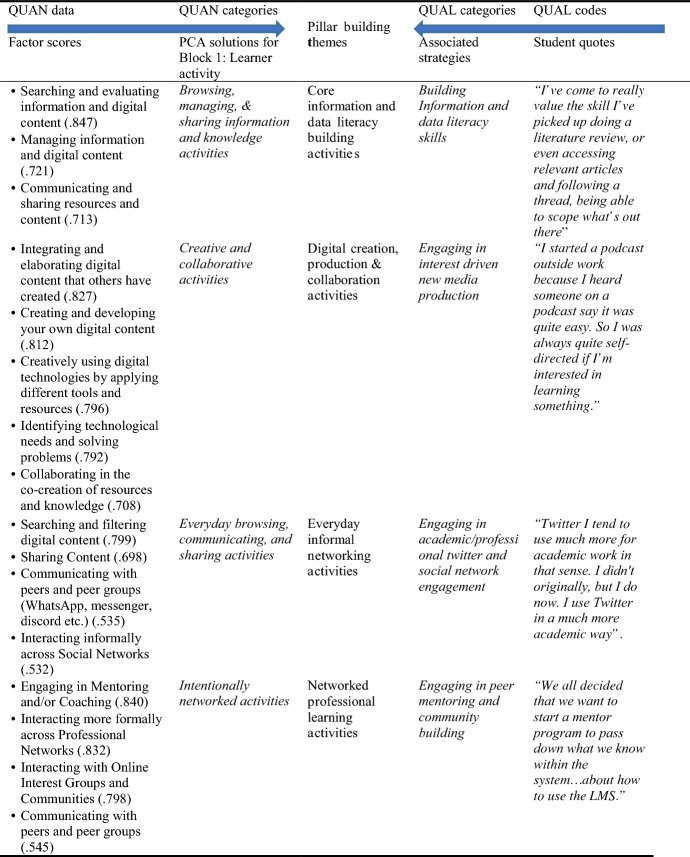
Learner activity sub-components

*PCA* principal component analysis, *QUAL* qualitative, *QUAN* quantitative

**Fig. 5:**
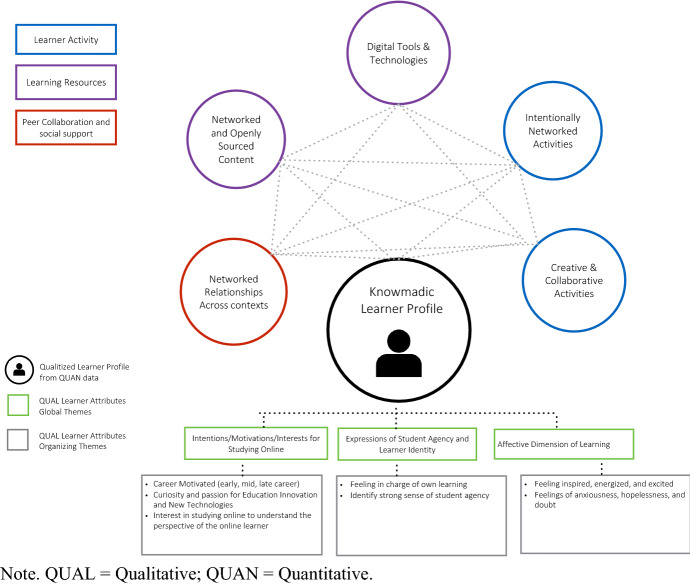
Integrated joint display of Knowmadic learner. *QUAL* qualitative, *QUAN* quantitative. *Source* Peters (2019)

The final joint display, presented in Fig. 5, used a qualitizing data transformation technique, where quantitative results are “transformed into a qualitative format that could be used for comparison with qualitatively accessed data” (Fetters et al., 2013, p. 2143). Scant MMR literature exists on the analytic process of converting quantitative data into data that can be analyzed qualitatively (Creamer, 2018). We followed the qualitizing process explained by Onwuegbuzie et al. (2011) which involved developing a narrative profile by creating narrative descriptions from numeric data and then comparing and integrating this data with qualitatively accessed data (Fetters et al., 2013). The below joint display presents a narrative profile of a *Knowmadic learner*, one of four profiles generated through hierarchical cluster analysis of survey data (Peters, 2019) to qualitize the quantitative data, something which has rarely been done in the EdTech literature. This approach follows an integration strategy at the interpretation and reporting levels. Qualitizing a learner profile from survey data (as displayed in the Knowmadic learner profile and associated blue, purple and red categories) and subsequently mapping it onto qualitative data is both a skill and art. It requires access to the most recent body of knowledge in the EdTech field and associated theoretical and conceptual considerations and debates, resulting in the eventual conceptualization of meaning to numerical results, as presented in the visual display in Fig. 4.

### Phase 3: Visual joint display iterations and final integrated meta-joint display model

**Fig. 6:**
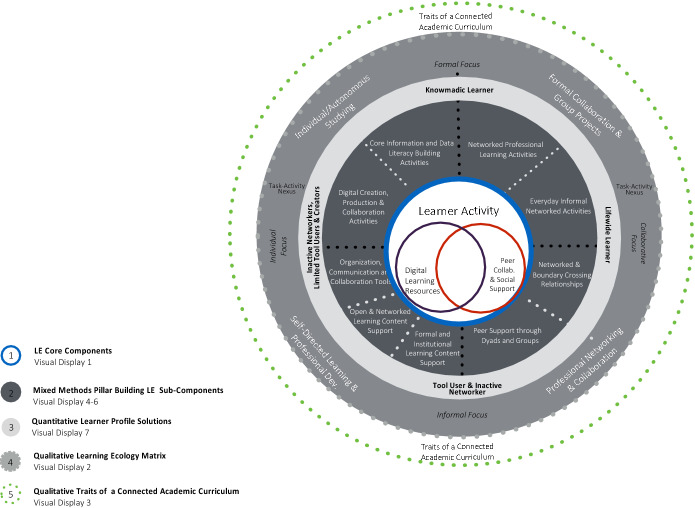
Meta-joint display of a model of student learning ecologies in online higher education. *Source* Peters et al. (2021)

A final meta-joint display is presented in Fig. 6. It represents a model of student learning ecologies in online higher education. After rigorous intra-method analysis was conducted, we identified a logical framework and visual representation for our display. A radial display was chosen as conducive to representing an expansive range of findings at different levels for a range of purposes. It also reflected well an ecological metaphor, including the integrated and dynamic process of contemporary learning, as well as the components and boundaries involved in learning across multiple contexts and practices. Informed by Haynes-Brown and Fetters (2021), joint display effectiveness can be assessed on three criteria, namely, *organizational intent, analytic intent,* and the *effectiveness of the visual presentation*. Using case-study and survey data, the organizational intent was to depict the ecological and integrated nature of activity-driven learner experiences in online postgraduate education. Organizationally, a radial display was chosen to optimally represent the components and contexts of an individual’s learning ecology. Important considerations at this stage were the layers and radii used to depict the contexts—from formal to informal and individual to collaborative—and the components of learner activity, including the material resources and relationship interactions relied on.

The *analytic intent* was to examine the interconnections and relationships between the different components and contexts of an individual’s LE by evaluating the extent to which the quantitative data supported the qualitative findings. The final meta-joint display analytically organized findings at five distinct levels of the model, beginning with the inner level: (1) the LE core components; followed by (2) the associated MMR pillar building sub-components; followed by (3) learner profile solutions derived through *qualitized* data transformation; followed by (4) the LE matrix which represents four conceptual zones of learning in the context of online higher education, and finally (5) the traits of a connected academic curriculum.

When constructing the visual joint display, consideration was made for coherence between the components and contexts of an individual’s LE. It was logical, for example, to place learner activity at the centre of the model. Consequently, it was logical to place the traits of the academic curriculum as a mediating factor at the outer border of the display, influencing an individual’s experiences of learning in postgraduate online education, including boundary-crossing learner activity across formal and informal and collaborative and individual contexts. The analytic intent aimed to develop a cohesive narrative about the student experiences of learning in online postgraduate higher education through a visual representation. For researchers new to visual joint display development, this can be accomplished by thinking creatively and visually about your integrated results throughout the research lifecycle, identifying a logical framework for organizing the two types of data together, and iteratively considering the most effective way of presenting each type of data while using software like PowerPoint or Illustrator to support the visual design.

We likewise evaluated the *effectiveness of the visuals* presented through a radial display looking for an ideal representation of the integrated findings. For instance, we aimed for a final integrated display which was self-explanatory and avoided unnecessary complexity while balancing detail and synthesis. The final integrated model was developed through four distinct iterations: (1) pre-publication initial model; (2) dissertation quality (Peters, 2019, p. 290); (3) peer-review quality (Peters et al., 2021, p. 7); (4) ETR&D methodological article. Design iterations were used to improve the effectiveness of the visuals by improving organizational and analytical intent. Core elements of each iteration include rigorous intra-method analysis and design features such as experimenting with different radial techniques, grayscale, color, and shapes to link information and summarize findings in a transparent way while avoiding complexity, following reporting guidelines presented in Table 1. In particular, between iteration 2 and 3, mixed analysis was included by creating pillar building techniques, while iteration 3 to 4 saw another mixed analysis procedure added (*qualitized* data transformation).

## Discussion and implications for practice

The aim of the current paper was to reflexively describe the process of creating a meta-joint display generated in a published study (Peters et al., 2021), linked to emerging methodological guidelines in the MMR literature. We have illustrated a range of procedures and discussed the practical complexities of MMR integration when using visual joint displays as an analytic tool for data interpretation and reporting, particularly relevant for EdTech researchers. We hope the guidelines can be extended to further use cases in the EdTech field and that implications of this manuscript can extend beyond the illustrative example presented. We suggest a distinction should be made, as others have done, between joint displays created for the purpose of reporting and those used as a tool for mixed analysis (Creamer & Edwards, 2022). We have aimed to extend what is already known in both the MMR and EdTech literature by offering step-by-step guidance on the process of visual joint display construction, illustrating how we integrated meta-inferences previously generated from a series of interlinking displays, while demonstrating the expansive range of purposes served by visual joint display construction to integrate at the literature review, theoretical, research design, interpretation, and reporting levels. The process highlights the close relationship between research design, selection and operationalization of integration strategies and the possibilities of novel and creative joint display development through mixed analysis.

Offering guidelines for research practice addresses the challenge of how to approach complex integration procedures by bringing methodological and reporting transparency to the process. Following guidelines and examples in the substantive literature can help researchers improve interpretations and reporting of investigated phenomena, add more depth and texture when answering research questions, and gain deeper insights that would not have been revealed otherwise. Below, in Table 3, recent methodological considerations by Fetters and Guetterman (2021) have been analyzed in light of how they have been applied in the current article.

**Table 3:** Checkbox of considerations when constructing a joint display for mixed data analysis

Considerations when constructing a joint display	Application in current study
1. Think creatively to build and expand upon other data combining strategies	• Visual mapping memoing were used at all stages of the research design lifecycle, from conceptualization, to analysis, and reporting
2. Conduct rigorous intra-method analysis	• Pillar-building technique and *qualitizing* data transformation was used
3. Develop a data sources table	• Several data source tables and displays were developed using SPSS, Microsoft PowerPoint, and tables in Microsoft Word
4. Become fully familiar with the results of both types of data	• Data familiarization occurred through research team meetings and presentations
5. Identify commonality of constructs and themes across the two types of data	• An exploratory sequential design underpinned by a robust theoretical model allowed for commonality and coherence between themes and constructs
6. Identify a logical framework for organizing the two types together	• Radial display was chosen, influenced from the substantive literature and sensitizing model
7. Develop a preliminary display using the organizing framework chosen	• A preliminary display was developed to organize core constructs and components of the study
8. Iteratively consider the most effective way of presenting each type of data together based on how the data appear together in the display	• From preliminary display to final iteration, intra-method techniques were used to assess the most effective way of presenting MMR findings
9. While building the display, consider not only the appearance but the implications of the findings	• Meta-joint display for analysis and reporting allowed gaining deeper insights that would not have been revealed otherwise, guiding interpretations of the findings, and shaping the reporting of results, and implications of the study
10. Consider adding color, grayscale, lines, overlap of symbols/shapes or some other mechanism to link information	• Color, grayscale, circular shapes in layers based on a radial design to link different information
11. Consider options for data transformation, including intra-method transformation and cross method data transformation	• Data transformation was used through *qualitized* findings of learner profiles
12. Consider if you have multiple joint displays whether you can create an effective meta-joint display figure	• A meta-joint display was built from seven distinct visual displays
13. Consider the extent the display will appeal to and be easily understood by the intended audience	• A radial design was chosen to link information and summarize findings, and the meta-joint display was piloted in research seminars and conferences to infer whether the display was appealing and easily understood
14. Consider again the implications of the two types of data	• Meta-joint display development as *process* and *product* was continuously used to shape mixed insights and interpretations, including on a methodological and empirical level

*Source* Fetters and Guetterman (2021)

We faced several challenges in the process of integrating findings through visual joint display development. Although following practical strategies to determine how and why to integrate can mitigate such challenges (Fetters et al., 2013), as a new researcher to mixed methods integration, there was an overwhelming sense of uncertainty and doubt when beginning initial joint-display development. The first challenge was developing a transparent research design which leads to rigorous intra-method and mixed analysis procedures at the interpretation and reporting levels. The second series of challenges related to the analytical and organizational intent, and the effectiveness of the visual display. We overcame such challenges in the current study through iterations at different publishing stages in the research process. Reviewing empirical examples and methodological guidelines helped overcome these challenges as we found inspiration in illustrative examples. A final challenge relates to publishing restraints, including the need to justify to editors and reviewers who are not familiar with the techniques or opportunities of visual joint displays as analytical tools. Methodological transparency was essential here, so following guidelines for mixed analysis and reporting is recommended.

### Visual joint displays as emergent technique in EdTech research

It is important to discuss the emergent and creative character of visual joint displays and mixed analysis, especially in the EdTech field where few empirical examples exist. Although guidelines are important for consideration, and are increasingly common for informing MMR for an expansive range of purposes (Creamer & Edwards, 2022; Fetters & Guetterman, 2021; Guetterman et al., 2021), the process we followed for joint display development also involved an inventive and serendipitous dimension that can only be achieved when there is room for unplanned experimentation and innovation. Leaving room for such processes allowed emergent and unexpected results to be generated. Unexpected and novel visual joint displays are difficult to fully plan beforehand, especially when limited previous examples exist in the empirical literature, and when few educational research methods textbooks cover such techniques. In particular, when conducting integration procedures in our study, we remained open for inspiration, including attending webinars and training courses which can offer valuable sources of information and methodological strategies.

### Limitations

A potential limitation of visual joint display development as conducted in this study are the challenges associated with advanced MMR designs and theory-driven and theory-advancing EdTech research. Responding to this challenge requires methodological, epistemological and theoretical coherence and clarity throughout all phases of the research lifecycle. There is a need to ensure a consistent alignment between theoretical frameworks and ontological definitions, research design, integration procedures, and the purposes and intentions of joint visual display construction. Another challenge in this article is aligning the integration guidelines followed throughout our procedural diagram (Fig. 2), and the emergent and creative character of joint display development, leaving room for unplanned and serendipitous creativity during joint visual display iterations.

## Conclusion

As the application of MMR research designs become increasingly relevant for addressing the complex problems that arise in teaching and learning environments in higher education, there is a need for transparent methodological guidance on visual joint display construction as a *process* of mixed analysis and as a final integrated *product* suitable for summarising findings when reporting. MMR studies which demonstrate explicit integration in the EdTech field are scarce, and even fewer apply integration strategies recommended in the literature, such as visual joint displays. Failure to address the challenge of comprehensive integration may result in missed opportunities for deeper insights or for pushing the field forward through theory-driven and theory-advancing research. To address this methodological problem, we used an illustrative research design to extend what is already known about mixed analysis and visual joint display for advancing research in the EdTech field by demonstrating how to integrate previously generated findings and meta-inferences from a series of interlinking displays. We were able to illustrate the expansive range of purposes served by visual joint display construction to integrate at the literature review, theoretical, research design, interpretation, and reporting levels.

## Data Availability

The data that support the findings of this study are available on request from the corresponding author [M.P.].
